# Tumor growth and vascular redistribution contributes to the dosimetric preferential effect of microbeam radiotherapy: a Monte Carlo study

**DOI:** 10.1038/s41598-024-77415-5

**Published:** 2024-11-04

**Authors:** Ramon Ortiz, José Ramos-Méndez

**Affiliations:** Department of Radiation Oncology, University of California San Francisco, 1600 Divisadero Street, San Francisco, CA 94143 USA

**Keywords:** Microbeam radiotherapy, Spatially fractionated radiotherapy, Vasculature, Computational modeling, Radiotherapy, Biological physics

## Abstract

The radiobiological mechanisms behind the favorable response of tissues to microbeam radiation therapy (MRT) are not fully described yet. Among other factors, the differential action to tumor and normal tissue vasculature is considered to contribute to MRT efficacy. This computational study evaluates the relevance of tumor growth stage and associated vascular redistribution to this effect. A multiscale approach was employed with two simulation softwares: TOPAS and CompuCell3D. Segmentation images of the angioarchitecture of a non-bearing tumor mouse brain were used. The tumor vasculature at different tumor growth stages was obtained by simulating the tumor proliferation and spatial vascular redistribution. The radiation-induced damage to vascular cells and consequent change in oxygen perfusion were simulated for normal and tumor tissues. The multiscale model showed that oxygen perfusion to tissues and vessels decreased as a function of the tumor proliferation stage, and with the decrease in uniformity of the vasculature spatial distribution in the tumor tissue. This led to an increase in the fraction of hypoxic (up to 60%) and necrotic (10%) tumor cells at advanced tumor stages, whereas normal tissues remained normoxic. These results showed that tumor stage and spatial vascular distribution contribute to the preferential effect of MRT in tumors.

## Introduction

Microbeam radiation therapy (MRT)^1^ is a form of spatially fractionated radiotherapy (SFRT) that uses arrays of micrometric (50 to 100 μm) kV X-ray planar beamlets, called microbeams, separated by a center-to-center distance of 200 to 400 μm. MRT lateral dose distributions are characterized by a succession of high-dose areas, called peaks, followed by areas of low dose, called valleys. Preclinical investigations have shown a significant increase in normal tissue radio-resistance as compared to seamless irradiations. For instance, an increase in the tolerance dose up to 300 to 600 Gy to the brain tissue^2–5^, spinal cord^6^ and skin^7^ has been observed. Along with the reduction of normal tissue toxicity, MRT has been shown to lead to a similar or increased survival and tumor regrowth delay as compared to conventional radiotherapy techniques^8–11^. A comprehensive summary of MRT studies can be found in the literature^12^.

The description of the radiobiological mechanisms behind this favorable response is still incomplete and cannot be solely explained by direct cell lethality^13^. Biological mechanisms that may be triggered by this highly heterogeneous irradiation pattern are the following: cell signaling and bystander effects, radiation-induced immune response, stem cell migration and proliferation, and differential vascular action to the normal and tumoral tissues^14^. The present study focuses on the differential vascular effect. Several studies showed that the MRT induced damage depends on the stage of vascular maturation^15,16^. Mature vasculature showed negligible perfusion effects after MRT irradiation whereas large unperfused areas were observed at earlier stages of the vasculature development^17^. This phenomenon is typically associated with the complete pericyte coverage of mature vessels as compared to immature capillary, which provides an increased radiosensitivity^17^ and rapid reparation of vascular endothelium^4^. The larger fraction of immature vasculature in tumor tissues is considered to be related with the MRT-induced increased capillary rarefaction and inter-vessel distances, and the decrease in the number of vessels and blood perfusion in tumors^11,18^, as compared to the relatively unchanged perfusion, blood volume, or vascular density in normal tissues^15,16^.

Within this context, the aim of this work is to evaluate whether the spatial redistribution of vasculature in response to the tumor growth further contributes to the differential effect of MRT in tumor and normal tissue vasculature. We evaluate the differences in oxygen perfusion and cell outcome derived from the oxygenation levels in normal tissues and different stages of the tumor growth exposed to MRT. Multi-scale Monte Carlo computational modeling simulations were employed to simulate the physical action of MRT irradiation and the individual subsequent biochemical and biological processes in controlled conditions.

## Materials and methods

### Software

In this work, two Monte Carlo (MC) toolkits were used: TOPAS^19,20^ for the computation of dose distributions, and CompuCell3D^21^ for the simulation of cell behavior and biochemical field diffusion. TOPAS, based on the Geant4 MC toolkit, is a freely available and open-source platform for the simulation of radiation-matter interactions dedicated to basic and applied research in medical physics. The TOPAS version used in this work was OpenTOPAS version 4.0, available on the TOPAS collaboration GitHub (https://github.com/OpenTOPAS). This version of the TOPAS code is a continuous development from TOPAS version 3.9. CompuCell3D (CC3D) is a modeling environment that allows the Monte Carlo simulation of cell and tissue-level interactions using the Glazier-Graner-Hogeweg (GGH) approach^22,23^. In this software the spatial and temporal scales are defined in terms of voxels and MC steps (MCS). The CC3D version employed in this work was v.4.2.5. The TOPAS-Tissue framework, validated in a previous work^24^, was used to interface both software.

### Vasculature geometry

The angioarchitecture of a CD1-E mouse brain was used. Segmentation images of the vasculature of the whole mouse brain were taken from the VesSAP database^25,26^ (publicly available at https://github.com/vessap/vessap). The resolution of these images is 3 × 3 × 3 µm^3^. In the present study, we considered a 0.7 × 0.7 × 0.7 mm^3^ region at the center of the right brain hemisphere. As described in Section 2.3, periodic boundary conditions were applied to avoid edge effects in the diffusion of the chemical fields simulated. The lattice was resampled to a grid of 6 × 6 × 6 µm^3^ voxels for the sake of improving the efficiency of simulations. This resizing was assumed not to decrease the detail of vasculature segmentation since the minimum diameter of vessels in the original segmentation image was 6 μm. The cropping and resizing of the original geometry were performed using a validated tool for the creation of biological geometries for computational modeling in radiobiology, described elsewhere^27^. The use of this tool ensured that the fraction of volume occupied by the vasculature was equivalent in both, original and resampled geometries (14.6% and 14.3%, respectively). This geometry was uploaded to CC3D for simulation. To remove potential artifacts in the capillary geometry due to the lattice resizing (e.g., small capillary discontinuities), we simulated the vascular modeling process in CC3D. For that, we assumed the autocrine secretion of a chemoattractant and vascular cell elongation as the main actors in the vascular architecture modeling^28^. We considered a VEGF-like field secreted by vascular cells toward the medium with a diffusion coefficient of 10 µm^2^/s and a decay constant of 0.65 h^−1^^29^. Cell elongation was modeled by assigning a target major axis length. This value was 20 pixels to obtain a mean vascular cell length within experimentally measured ranges of 124 ± 7 μm^30^. The mean cell volume was 48.8 ± 2.1 voxels (10540 ± 454 µm^3^), which is within the vascular cell dimensions reported in the literature^31,32^. As described in previous works, adjacent voxels containing vascular capillaries were assigned to the same cluster forming the beforementioned vascular cells using a validated clustering tool for creation of biological geometries^27^ This vasculature geometry was considered the *normal tissue vasculature*.

To generate a *tumor-induced vasculature*, i.e., remodeling of the normal tissue vasculature due to tumor growth, we simulated the growth of an avascular tumor in CC3D. The model by Shirinifard et al.^33^ was adopted as follows. A single tumor cell was placed at the center of the lattice. Tumor cell behavior was modeled by their level of oxygenation, i.e., oxygen partial pressure (pO_2_). The pO_2_ in vascular cells was 90 mmHg. From vascular cells, oxygen diffuses to the cell lattice and tumor cells increase their volume depending on their pO_2_ concentration following a Michaelis-Menten behavior. Tumor cells have an average volume of 3375 µm^3^ and divide when they reach a volume of 6750 µm^3^. A detailed description of this model can be found in the literature^33^. In the present study, no angiogenesis was simulated to maintain the same number of vascular cells (i.e., the same maximum capacity of blood volume) as in the normal tissue vasculature scenario. Indeed, the early stages of tumor development (up to 1 mm diameter tumor spheroids, as the one considered in this study) are avascular^34^. The vascular and tumor cell configurations were recorded at different stages of the tumor growth (up to 20 days). Hereafter, these stages are denoted as the letter *D* followed by the day of growth (e.g., *D20* for day 20). The dimensions of the lattice allowed the tumor growth to a maximum diameter of 700 μm, which corresponds to typical sizes of tumor spheroid used in radiobiology research^35^.

The spatial distribution of the vascular cells in the lattice was evaluated by the standard deviation of the local density of vascular cells in lattice subregions. This is, the entire lattice was divided in subregions and the density of vascular cells in each subregion was computed as the number of voxels occupied by vascular cells divided by the volume (in pixels) of the subregions. Then, the standard deviation of the local densities was computed. A higher standard deviation of local densities indicates a lower uniform distribution of vascular cells. Hereafter, the increase in standard deviation of local densities is referred to as the decrease in spatial uniformity of vasculature.

### Oxygen diffusion

The level of oxygenation was modeled with CC3D by the partial pressure of oxygen (pO_2_) in cells. To simulate the flow of oxygen through capillaries, a diffusion field was used. The oxygen field in vascular cells was denoted *vascular pO*_*2*_. The diffusion coefficient in medium and non-vascular cells was nil, restricting its diffusion to vascular capillaries. This ensures that only the vascular pO2 field is transported (i.e., diffused) through boundaries of voxels containing vascular cells, mimicking the flow of oxygen through capillaries. That is, this field does not diffuse from voxels containing vascular cells to other lattice voxels not conforming to the vasculature. The diffusion of oxygen from the vasculature to the rest of the tissue is modeled by the cellular pO2 field, described in detail hereafter. To create a diffusion direction, i.e., simulate the field supply or drainage, two new cell types were defined: donor and acceptor cells. Donor and acceptor cells were placed at every interface of vascular capillaries with the six sides of the simulation volume borders, i.e., three sides containing donor cells and the three opposite sides acceptor cells. A constant vascular pO_2_ concentration of 90 mmHg was assigned to donor cells to mimic the arterial partial pressure of oxygen^36^, whereas for acceptor cells was nil to allow the field flux whenever donor and acceptor cells are connected by vascular cells. The value of the vascular pO_2_ field in each vascular cell was defined by its diffusion though the vascular capillaries, which is dependent on the size of vascular capillaries. In addition to the vascular pO_2_ field, a *cellular pO*_*2*_ diffusion field was defined. This field was allowed to diffuse within the entire cell lattice. At each MCS, the field value in vascular cells was updated to be equal to the vascular pO_2_. From there, this field diffuses to the rest of the cell lattice with its spatial and temporal evolution dictated by its diffusion coefficient. The diffusion coefficient of pO_2_ field in bulk tissue was set to 2 × 10^3^ µm^2^/s^37^. Periodic boundary conditions were applied to the diffusion of the cellular pO_2_ field to avoid edge effects. That is, at the volume boundaries, the field diffused through the boundary and re-entered the volume through the opposite boundary to preserve continuity.

**Figure 1:**
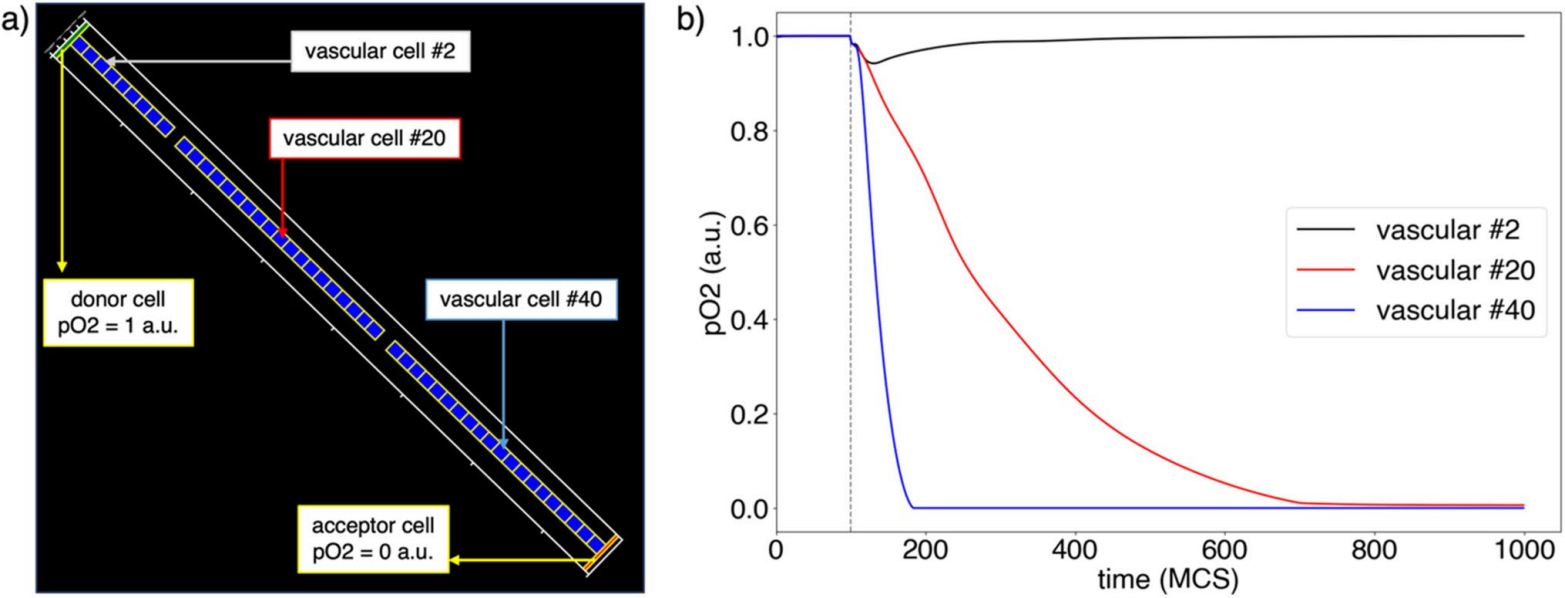
Temporal evolution of vascular pO_2_ after vascular cell death. (**a**) Representation of cell configuration after death (MCS = 100). (**b**) Temporal evolution of vascular pO_2_ in the different cells shown in (a). The dotted vertical line shows the moment cells #10 and #30 are removed from the capillary (time = 100 MCS).

The temporal and spatial evolution of the vascular pO_2_ field was dependent on its diffusion within vascular cells and the amount of vascular pO_2_ that diffuses away from vascular cells in the form cellular pO_2_. In our simulation, this was modeled as follows. The difference in cellular pO_2_ at each MCS with respect to the previous MCS in vascular cells was evaluated to determine the concentration of pO_2_ drop due to diffusion to the medium. Then, the value of vascular pO_2_ in vascular cells was the result of the combination of vascular diffusion from a donor cell and the pO_2_ change due to diffusion to the medium in the form of cellular pO_2_. Overall, this model ensured that when the equilibrium was disrupted (e.g., when vascular cells were removed from the lattice due to radiation-induced necrosis) one of three scenarios occurred: (1) vascular cells only connected to donor cells had a constant vascular pO_2_ value in equilibrium due to its constant supply by diffusion, (2) vascular cells only connected to acceptor cells reduced their vascular pO_2_ due to drainage by diffusion and cellular pO_2_ diffusion to the medium, (3) vascular cells not connected to donor or acceptor cells (i.e., no supply or drainage by vascular blood flux) dropped their oxygen concentration due to the diffusion of pO_2_ to the cell lattice. These three scenarios are shown as a proof-of-concept example in Fig. 1a. This example consisted of 50 vascular cells organized in a single capillary, with a donor and acceptor cells with vascular pO_2_ values of 1 and 0 at the sides. The initial pO_2_ value in vascular cells was set to 1 a.u. Two of these cells (#10 and #30) were removed from the capillary at MCS = 100. The temporal evolution of the vascular pO_2_ field was evaluated. As shown in Fig. 1b, from MCS = 100, vascular cells in scenario (2) reduced their concentration more rapidly than cells in scenario (3). This was due to the combined action of drainage and cellular pO_2_ diffusion in scenario (2), whereas only cellular pO_2_ diffusion contributed to the field reduction in scenario (3). Cells in scenario (1) maintained their vascular pO_2_ level, equal to their initial concentration (i.e., constant concentration in the donor cells).

### MRT irradiation and cell damage

MRT irradiations (one fraction) were performed at the different stages of the tumor proliferation (from D0 to D20). Longer times than D20 were not simulated since the increase in the number of tumor cells as the tumor proliferates would cause the cell lattice to be overcrowded, leaving no space for the system to evolve in terms of cell division, vascular morphological changes and preservation of the biological constraints. In our CC3D simulations, longer times than D20 led to artifacts, for instance the tumor not growing due to the lack of free space in the simulation volume, influencing the results of the study.

**Figure 2:**
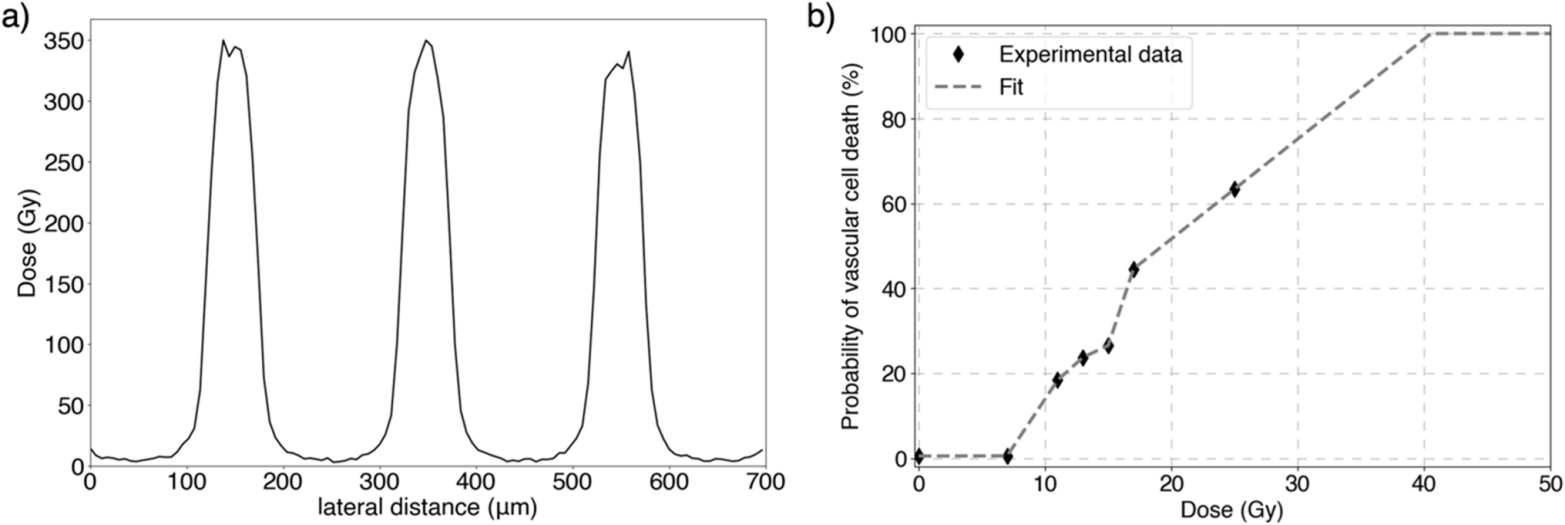
(**a**) MRT dose profile at the center of the scoring volume, and (**b**) probability of vascular cell death as a function of the absorbed dose. Experimental data from^41^.

MRT dose distributions were simulated using TOPAS. The irradiated configuration considered consisted of three 50 μm-wide and 1.2 mm-long planar microbeams separated by a center-to-center distance of 200 μm, as typically used experimentally^38^. The kV X-ray energy spectrum and lateral beam divergence (0.5 mrad) of the biomedical beamline of the European Synchrotron Radiation Facility (ESRF) (ID17, Grenoble, France) in microbeam mode were used^39^. The dimension and resolution of the scoring volume mimicked the cell lattice used in CC3D, i.e., 0.7 × 0.7 × 0.7 mm^3^ size with 6 × 6 × 6 µm^3^ voxels. The scoring volume was composed of water it was and placed at 5.5 mm depth in water to mimic a typical tumor implantation depth in mice^40^. The range cut for all particles was set to 1 μm. The dose profile was normalized to common experimental doses, i.e., 350 Gy peak dose^38^ (see Fig. 2a). The statistical uncertainty in dose distributions, defined as the mean uncertainty in voxels with at least half of the maximum dose (i.e., peak dose), was 2.5%.

The action of radiation on the vasculature was modeled by the death/survival of vascular cells after the MRT irradiation. Vascular cells receiving doses between 0 and 25 Gy were considered dead with a probability given by the microvascular endothelial apoptosis dose response measured by Garcia-Barros et al.^41^. This model was derived considering conventional dose rates. The consideration of higher dose rates is discussed in detail in Section 4. The death probability at doses higher than 25 Gy was extrapolated from the two highest experimental dose points. MRT dose distributions are mainly characterized by peak and valley doses and high dose gradients. As a result, most of the cells received relatively low valley doses that are within measured values (0 to 25 Gy)^41^, or very high ablative peak doses (> 50 Gy), 58% and 37% of cells, respectively; and only a small proportion of cells (5%) receive dose values in the range of extrapolation. Thus, uncertainties associated with the dose extrapolation have a small effect on the results of this study. Figure 2b shows the probability of vascular cell death considered in this study as a function of the dose received. The mean dose of the voxels conforming to each cell was considered for this evaluation. Dead vascular cells were removed from the lattice in the CC3D simulation.

Since the action of radiation in MRT on normal and tumor tissues is not fully modeled yet and cannot be only described by the DNA damage^13^ the action of radiation on non-vascular cells was considered as follows. Cells at the microbeam path, i.e., within its dose FWHM, were removed from the cell lattice to reproduce the *strip*-like regions of the size of the microbeam width of loss of neuronal and glial cells observed in in vivo murine models^42^ due to the ablative peak doses (350 Gy) used. The oxygen enhancement effect in cell death as function of the level of oxygenation was not explicitly modelled since, at the time of irradiation, most of cells in the lattice were normoxic.

### Evaluation of oxygen depletion and cell outcome

To evaluate the effect of MRT on tissue oxygenation, we modeled the cell outcome based on their partial oxygen pressure (pO_2_). For that, we simulated the uptake and levels of pO_2_ in individual cells in a lattice mimicking the cell density in the cortex of a mouse brain, i.e., 127,870 cells/mm^3^^43^. The mean normal (non-tumor) cell volume was ~ 1944 µm^3^, as for neuroglial cells^44^. In the tumor-induced scenarios, the arrangement of vascular and tumor cells was modeled as described in Section 2.2. The pO_2_ consumption was modeled as described in Section 2.2 with a maximum uptake per cell of 0.6 mmHg/s (assuming a murine cerebral oxygen consumption of 1.97 µmol/g/min^45^, a brain density of 1.04 g/cm^3^, and an equivalence between pO_2_ and oxygen concentration of 100 mmHg/150 µM^46^). The oxygen uptake of tumor cells was set to ten times higher than for normal tissue cells^32^. Cells with pO_2_ levels lower than 5 mmHg were considered hypoxic^47,48^ and cells with less than 1 mmHg as necrotic (dead by low levels of oxygen supply)^48^. Hypoxic cells were allowed to transition to normal cells when pO_2_ increased to values higher than 5 mmHg. Oxygen diffusion was simulated pre- and post-irradiation as specified in Section 2.3. The numbers of normoxic, hypoxic, and necrotic cells were scored in the normal tissue and tumor scenarios. The scenario pre-irradiation was considered the *reference* scenario.

## Results

### Normal tissue and tumor vasculature geometries

**Figure 3:**
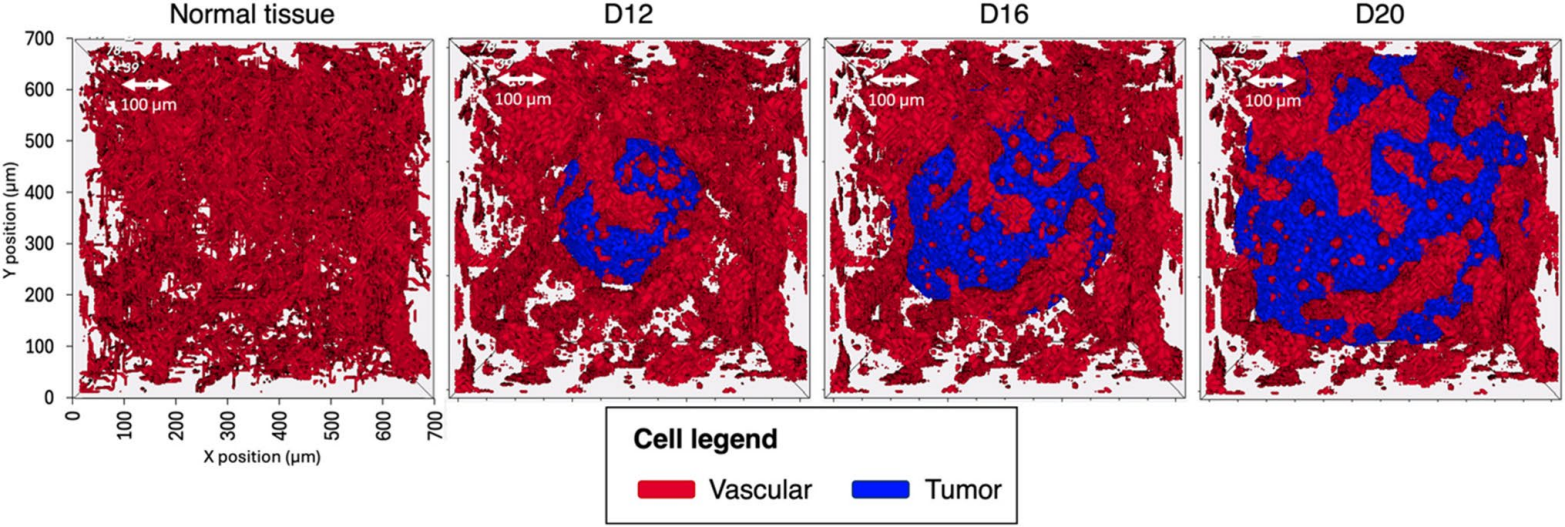
3D visualization of the normal tissue and tumor (D12, D16, and D20) geometries. For the sake of visualization, the normal tissue cells are not shown.

Figure 3 shows the geometries, including vascular and tumor cells, in the normal tissue and various tumor scenarios. D12 to D20 scenarios are presented as they are considered representative of the evolution of vasculature and tumor growth. Shorter times (prior to D12) behave similarly to the normal tissue scenario since the size of the tumor is still small to influence the spatial vascular redistribution. In the normal tissue scenario, vascular cells cover a 14.8% of the lattice volume. In tumor scenarios, a similar fraction of volume is observed (see Table 1). The tumor volume ranges from 0.019 mm^2^ (D12) to 0.195 mm^2^ (D20), as specified in Table 1. The remaining lattice volume is filled by normal tissue cells.

The uniformity of the spatial distribution of vascular cells (i.e., standard deviation of local densities, see Section 2.2) decreases with the stage of tumor proliferation. The decrease in spatial uniformity with respect to the normal tissue scenario rises from 6.2% at D12 to 18.5% at D20 (see Table 1). This is caused by the displacement and accumulation of vascular cells towards the tumor contour (i.e., away from the lattice center, where the center of mass of the tumor is located) as the tumor grows.

**Table 1:** Percentage of lattice volume occupied by vascular cells, tumor volume, and decrease in spatial uniformity of vasculature in the normal tissue and tumor scenarios (D12, D14, D16, D18, and D20). The decrease in the uniformity of the spatial distribution of vascular cells in tumor scenarios is expressed with respect to the normal tissue scenario.

	Normal tissue	D12	D14	D16	D18	D20
V _vasc_ (%)	14.8	14.6	14.8	14.5	14.1	14.0
V _tumor_ (%)	-	5.5	13.5	27.6	46.5	56.5
Decrease in spatial uniformity of vasculature (%)	-	6.2	10.4	14.1	16.8	18.5

### Cell death after MRT irradiation

**Figure 4:**
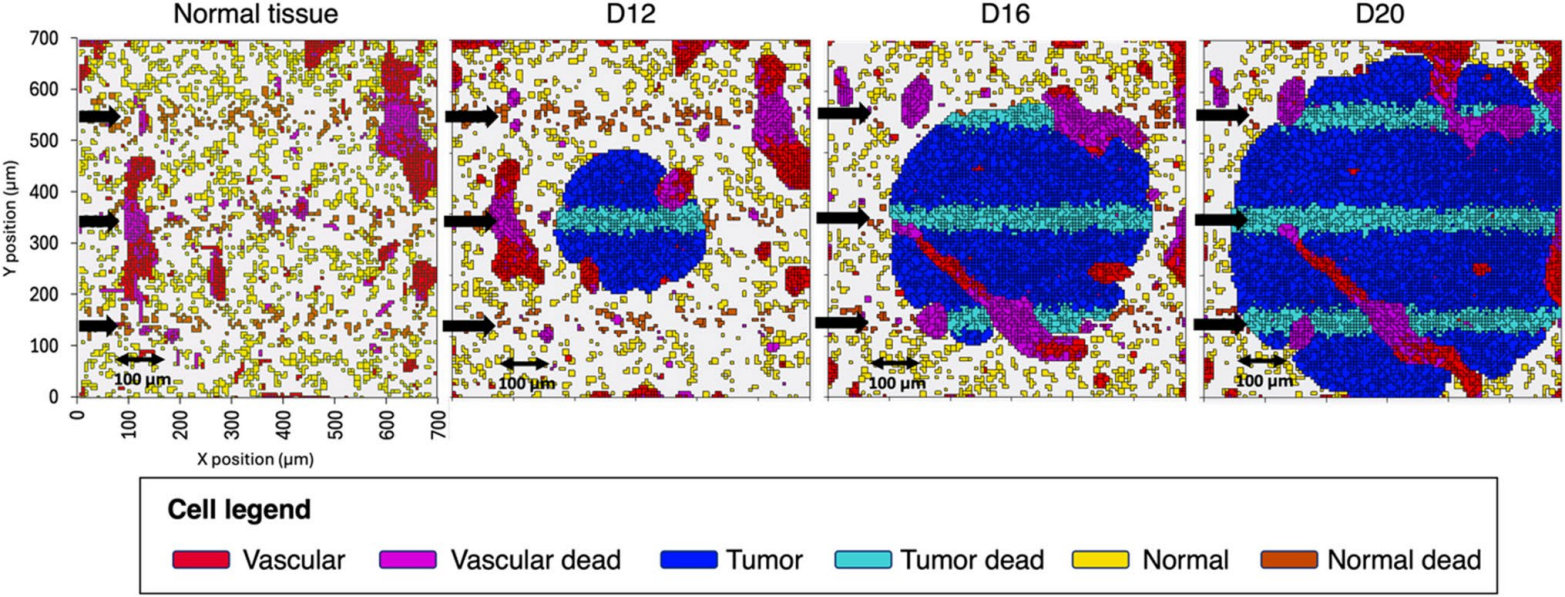
Visualization (2D slice at the center of the lattice) of the spatial distribution of intact and dead cells after MRT irradiation in normal tissue and tumor (D12, D16, and D20) scenarios. Black arrows indicate the direction of microbeams.

Figure 4 illustrates the action of the MRT irradiation, in terms of cell death, in the normal tissue and tumor scenarios. Peak and valley doses, computed at the center of the cell lattice, are 350 Gy and 6 Gy, respectively.

The fraction of dead vascular cells is nearly constant in all the geometries, accounting for 46.5% (5515 cells) and 46.0% (5410 cells) of the total number of vascular cells in the normal tissue and D20 scenarios, respectively (see Table 2). Similar fractions of normal/tumor inactivated cells are observed in all scenarios, ranging from 19.9 to 21.3% (see Table 2).

**Table 2:** Percentage of vascular and normal and tumor cells dead by the direct action of radiation.

	Normal tissue	D12	D14	D16	D18	D20
Dead vascular cells (%)	46.5	48.5	48.8	48.9	48.1	46.0
Dead tumor and normal cells (%)	19.7	21.3	19.9	21.1	21.0	21.2

### pO2 depletion and cell outcome

The levels of pO_2_ in the cell lattice in reference conditions (pre-irradiation) resemble experimentally measured levels in brain tissue. The mean pO_2_ in normal tissue cells in the normal tissue scenario was 26.7 ± 5.2% (1 SD), similar to physioxic levels reported in literature (33.8 ± 2.6)^49^.

**Figure 5:**
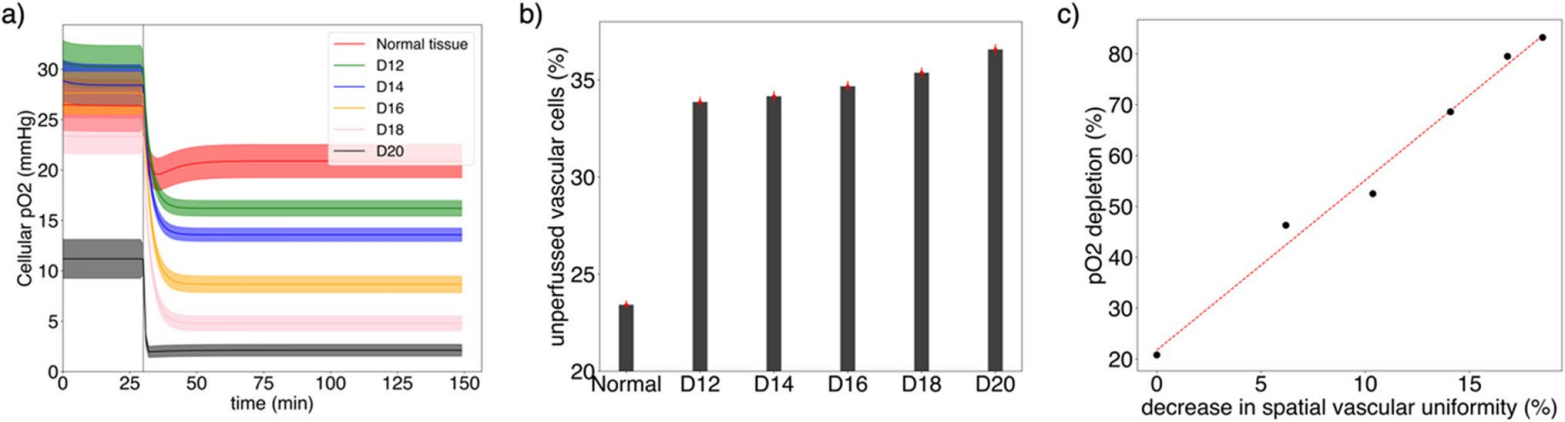
(**a**) Cellular levels of pO_2_ pre- and post MRT irradiation in normal tissue and tumor (D12, D14, D16, D18, and D20) scenarios. Solid lines indicate mean values, and the shadow areas correspond to one standard deviation. The vertical grey line indicates the time of the MRT irradiation (t = 30 min). (**b**) Fraction of unperfused vascular cells at the steady state after MRT in normal tissue and tumor scenarios. (**c**) Relationship between pO_2_ depletion after MRT irradiation (with respect to pre-irradiation) and the decrease in spatial uniformity of vasculature (expressed with respect to the normal tissue vasculature uniformity).

The pO_2_ depletion after MRT irradiation depends significantly on the stage of the tumor proliferation. At the steady state post-irradiation, the mean cellular pO_2_ in the cell lattice is 20.9 ± 3.3 mmHg, 16.2 ± 1.6 mmHg, 13.6 ± 1.3 mmHg, 8.7 ± 1.7 mmHg, 4.8 ± 1.5 mmHg and 1.9 ±1.2 mmHg in the normal tissue, D12, D14, D16, D18 and D20 scenarios (see Fig. 5a). This entails a relative depletion in pO_2_ of 20.8%, 46.3%, 52.5%, 68.6%, 79.5%, and 83.2% with respect to the pre-irradiation steady state. The detailed spatial distribution of the oxygen field across the cell lattice was not explicitly evaluated in this work. We considered the mean and standard deviation of the pO_2_ field value in the cell lattice as a quantity defining the global decrease in oxygenation assuming a nearly Gaussian distribution. On the other hand, a more detailed modeling of the spatial distribution of hypoxic and necrotic areas would show a slight skew depending on the specific scenario. In those cases, the cell density and cell uptake variations within the cell lattice should be considered.

The time evolution of pO_2_ from MRT irradiation to the steady state follows a characteristic Boltzmann diffusion equation behavior. On the other hand, the fraction of unperfused vascular cells (i.e., fraction of vascular cells where vascular pO_2_ is depleted) at the steady state after MRT increases from 23.4% in the normal tissue to 36.6% at D20 (see Fig. 5b). The number of unperfused vascular cells pre-irradiation was nil in all cases. Overall, for the tumor growth stages studied, the relative reduction in cellular pO_2_ after MRT irradiation appears to be linearly correlated with the decrease in uniformity of the spatial distribution of vascular cells at each tumor stage (see Fig. 5c).

**Figure 6:**
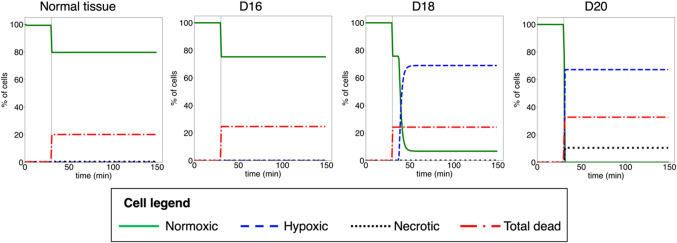
Fraction of normoxic, hypoxic, necrotic and total dead cells pre and post MRT irradiation in normal tissue and tumor (D16, D18, and D20) scenarios. The fraction of total dead cells is the sum of necrotic cells due to the low levels of pO_2_ and cell dead by the direct action of radiation. The vertical solid grey line indicates the time of the MRT irradiation (t = 30 min).

The pO_2_ depletion in the cell lattice after MRT irradiation has an impact on the fraction of hypoxic and necrotic cells, which also depends on the stage of the tumor-induced vasculature. As shown in Fig. 6, in normal tissue and early tumor stages (up to D18), all cells in the lattice maintain aerobic levels of pO_2_ (above 5 mmHg). However, MRT irradiations at more advanced stages of the tumor growth led to a significant fraction of hypoxic (69.0% and 67.2% at D18 and D20, respectively), and necrotic cells (10.5% at D20; <1mmHg). In the latter scenario, this contributes to a 10% increase in the fraction of inactivated tumor cells, besides the 20% contribution caused by radiation damage. The 20% increase in the fraction of dead cells at the time of irradiation (t = 30 min) displayed in Fig. 6 corresponds to radiation-induced inactivated cells (see Section 3.2).

## Discussion

Microbeam radiotherapy (MRT) is a technique that has shown favorable normal tissue and tumor responses in preclinical studies as compared to conventional techniques. However, the radiobiological basis of MRT is still not fully described. Among other mechanisms, the differential action on normal and tumor tissue vasculature is considered to contribute to this radiobiological response. This effect is mainly associated with the preferential action of MRT on immature tumor microvasculature as compared to mature normal tissue vessels. The objective of this work is to study whether the spatial redistribution of vascular cells at different tumor growth stages further contributes to the preferential vascular effect of MRT in terms of tissue oxygenation and cell outcome.

We found that the tissue oxygenation depletion after MRT irradiation depends on the stage of tumor proliferation at the time of irradiation, from 20% in the normal tissue scenario to more than 80% at late stages of tumor growth. Similarly, the fraction of unperfused cells (used as a surrogate for blood volume) increases by 13% from the normal tissue scenario to late stages of tumor proliferation. These results are in line with the experimentally measured decreased blood volume and local oxygen saturation in advanced tumors stages as compared to normal tissues^15,16,38^. This dependence was found to be correlated with the decrease of uniformity in the spatial distribution of vessels before irradiation. This decrease in uniformity as the tumor proliferates is associated to the accumulation of vascular cells towards the tumor contour. The number of vascular cells inactivated by radiation was nearly constant in all scenarios, i.e., the maximum capacity of blood volume pre- and post-irradiation was equivalent. These results suggest that the differential action of MRT on tissue oxygenation is also associated to the stage of tumor proliferation at the time of irradiation and the associated redistribution (i.e., decrease in spatial uniformity) of vasculature, besides the maturation stage of capillary observed in other studies^15,16^.

Analogously, the tissue outcome derived from oxygenation levels shows a dependence on the stage of tumor development. The tissue oxygen depletion after MRT does not have an impact on the reduction of normoxic cells for MRT irradiations of normal tissue and early stages of tumor growth. However, in the late stages of tumor development, a significant fraction (up to 60%) of tumor cells become hypoxic. These hypoxic areas within the tumor may contribute to slowing down the tumor proliferation, among several other effects described in the literature^50^. At more advanced tumor stages, a significant fraction of tumor cells (10%) become necrotic, increasing the proportion of dead tumor cells on the top of the ones inactivated by the direct action of radiation. These results agree with experimental observations of hypoxic and necrotic regions in tumors after MRT whereas normal tissues maintained physioxic oxygenation levels^15,38^. This suggests that this effect may contribute to the tumor control in MRT and that the efficacy in tumor control due to the damage of vasculature may increase in MRT treatments of advanced tumor stages. In addition, these results also hint that this effect may be more relevant in tissues with low oxygenation levels, where the transition to hypoxic/necrotic stages may occur at earlier stages of the tumor proliferation.

In this work, the increased radio-resistance of mature vessels as compared to immature capillary was not considered. This was chosen to study individually the potential dependence of the preferential effect of MRT on the spatial redistribution of tumor vasculature at different tumor development stages. Future works may consider both effects in combination to study their potential synergy to increase MRT efficacy. Those evaluations may include more complete cell response models specific to MRT, which are still missing nowadays, to complement the evaluation of radiation-induced vascular damage and repair and its implications in tissue oxygenation and outcome. Dose-rate effects were not considered in this work as our primary scope was to study the interplay between the spatial remodeling of vasculature as response to the tumor growth and the spatial dose delivery pattern in MRT. The potential differential effect of ultra-high dose rate (UHDR) delivery on cell response is still under research in the community. For instance, besides the differential action on cell response, the differential production of chemical species after UHDR irradiations^51^ may play a role in the induction of cell signaling effects, such as bystander effects, which are hypothesized to contribute significantly to the response of tissues to MRT^14^. Once established, the incorporation of such a response would complement the present study in future work.

## Conclusions

This work shows that the preferential action of MRT on tumor vasculature, in terms of decrease of tissue oxygenation and increased fraction of hypoxic and necrotic areas, is associated with the stage of tumor proliferation at the time of irradiation and decrease in the uniformity of the spatial distribution of vessels due to the tumor growth. This result contributes to advancing the description of the radiobiological mechanisms in spatially fractionated radiotherapy and, in particular, in microbeam radiotherapy.

## Data Availability

The data that support the results of this study are available from the corresponding author upon reasonable request.
